# High Diversity but Monodominance of Multidrug-Resistant Bacteria in Immunocompromised Pediatric Patients with Acute Lymphoblastic Leukemia Developing GVHD Are Not Associated with Changes in Gut Mycobiome

**DOI:** 10.3390/antibiotics12121667

**Published:** 2023-11-27

**Authors:** Sara Sardzikova, Kristina Andrijkova, Peter Svec, Gabor Beke, Lubos Klucar, Gabriel Minarik, Viktor Bielik, Alexandra Kolenova, Katarina Soltys

**Affiliations:** 1 Department of Microbiology and Virology, Faculty of Natural Sciences, Comenius University in Bratislava, 841 04 Bratislava, Slovakia; 2 Department of Pediatric Hematology and Oncology, Children’s Haematology and Oncology Clinic, Faculty of Medicine, Comenius University in Bratislava, 833 40 Bratislava, Slovakia; 3 Institute of Molecular Biology, Slovak Academy of Sciences, 841 04 Bratislava, Slovakia; 4 Medirex Group Academy n.p.o., Novozamocka 67, 949 05 Nitra, Slovakia; 5 Department of Biological and Medical Science, Faculty of Physical Education and Sport, Comenius University in Bratislava, 814 69 Bratislava, Slovakia

**Keywords:** multidrug resistance, gut microbiome, gut mycobiome

## Abstract

Graft-versus-host disease (GvHD) is a severe complication after hematopoietic stem cell transplantation (HSCT). Our study focused on identifying multidrug-resistant (MDR) gut bacteria associated with GvHD-prone guts and association with gut microbiota (GM) diversity, bacteriome, and mycobiome composition in post-HSCT patients. We examined 11 pediatric patients with acute lymphoblastic leukemia (ALL), including six with GvHD, within three time points: seven days pre-HSCT, seven days post-, and 28 days post-HSCT. The gut microbiome and its resistome were investigated using metagenomic sequencing, taxonomically classified with Kraken2, and statistically evaluated for significance using appropriate tests. We observed an increase in the abundance of MDR bacteria, mainly *Enterococcus faecium* strains carrying *msr(C)*, *erm(T)*, *aac(6′)-li*, *dfrG*, and *ant(6)-la* genes, in GvHD patients one week post-HSCT. Conversely, non-GvHD patients had more MDR beneficial bacteria pre-HSCT, promoting immunosurveillance, with resistance genes increasing one-month post-HSCT. MDR beneficial bacteria included the anti-inflammatory *Bacteroides fragilis*, *Ruminococcus gnavus*, and *Turicibacter*, while most MDR bacteria represented the dominant species of GM. Changes in the gut mycobiome were not associated with MDR bacterial monodominance or GvHD. Significant α-diversity decline (Shannon index) one week and one month post-HSCT in GvHD patients (*p* < 0.05) was accompanied by increased *Pseudomonadota* and decreased *Bacteroidota* post-HSCT. Our findings suggest that MDR commensal gut bacteria may preserve diversity and enhance immunosurveillance, potentially preventing GvHD in pediatric ALL patients undergoing HSCT. This observation has therapeutic implications.

## 1. Introduction

The human intestine possesses a complex ecosystem of trillions of microorganisms, i.e., the gut microbiota, which can turn into a place with specific pathophysiological conditions triggering the human immune system during hematopoietic stem cell transplantation (HSCT). One such condition, graft-versus-host-disease (GvHD), is a life-threatening complication of HSCT. During the development of GvHD, immunologically competent cells, especially donor T cells, are activated, leading to a response to recipient tissue antigens, and subsequently, human organs, including the hematopoietic system, the liver, and the intestine. The different histocompatibility complexes of donor and recipient, pretransplant chemotherapy, and gut radiation damage contribute to GvHD [1].

It is already an accepted fact that the intestinal microbiota has a significant impact on GvHD development. Studies on germ-free (GF) mice have shown a reduction in the risk of GvHD development in GF mice [2,3,4]. However, although progress in therapeutic advancements has significantly improved HSCT outcomes, multiresistant bacteria remain a challenge. The gut microbiota represents a reservoir of antimicrobial resistance genes. While imbalances in the gut microbiota are tightly associated with therapy and conditioning regimen, as well as parenteral diet, immune suppression leads to the colonization of resistant bacteria, often identified as the cause of bloodstream infections or sepsis [5]. 

Multiresistant bacteria, often referred to as multidrug-resistant (MDR) bacteria, are strains which have developed resistance to multiple antibiotics, limiting treatment options and increasing the risk of severe infections. Although the gut microbiota has been intensively studied in the context of HSCT treatment, studies investigating the gut resistome are lacking. Although some studies have provided information on the association of MDR colonization with increased bloodstream infection [6], others have not shown a significant impact on transplantation outcome [7]. Furthermore, studies on the loss of diversity and the expansion of resistant bacteria which colonize depleted environments and become dominant species in the gut are scarce. 

The enrichment of *Enterococcaceae*, *Staphylococcaceae*, *Streptococcaceae*, and *Pseudomonadota* was previously correlated with GvHD development. Moreover, *Prevotella* spp. or *Escherichia coli* are risk factors for GvHD [8]. In addition, GvHD is associated with deficiencies of *Lactobacillus johnsonii*, *Clostridium leptum*, and *Eubacterium rectale* in pediatric patients and with a lack of *Bacteroides* and *Parabacteroides* genera in adult patients [9,10]. GvHD development has also been associated with a low abundance of *Blautia* and *Akkermansia muciniphila* early post-HSCT [11]. 

Although the role of the gut mycobiome has been stressed in a few studies, to the best of our knowledge, there is no published evidence on its composition in pediatric oncology patients undergoing HSCT. Furthermore, no existing study has associated the dominance of multidrug-resistant bacteria with gut mycobiome composition and the implications for clinical outcomes. Understanding the dynamics of multiresistant bacteria in the context of GvHD is essential for the development of effective prevention and treatment strategies. 

We hypothesize that GvHD patients will demonstrate a unique microbial composition, characterized by lower diversity and increased abundance of resistant microorganisms within the gut microbiome. In this study, we aimed to analyze the gut bacteriome and mycobiome composition and the “gut profile” of resistant microorganisms. We focused on the association of the gut bacteriome and mycobiome and multidrug-resistant bacteria dominance within the gut of pediatric oncology patients with GvHD outcome after allo-HSCT. Finally, we investigated the diagnostic potential of the gut microbiome and resistome for GvHD development.

## 2. Results

### 2.1. Subject Characterization

**Figure 1:**
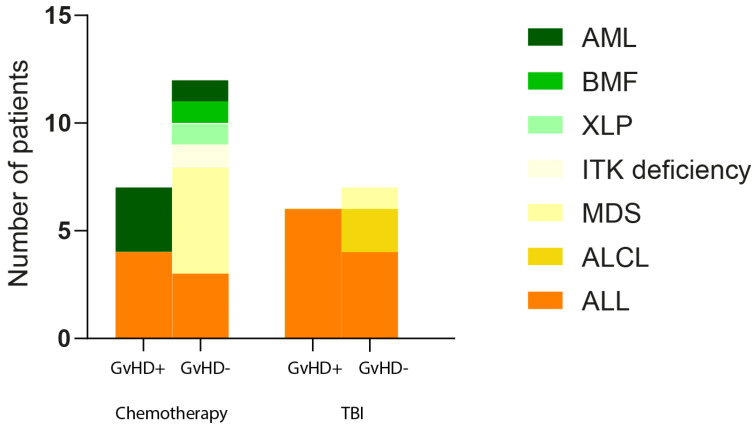
Graphical visualization of patients according to chemotherapy or TBI; groups with or without GvHD development. Different colors represent individual diagnoses.

We made a deep gut microbiome analysis of pediatric oncology patients (32) based on the therapy, age, BMI, sex, and diagnosis and determined the associations between established parameters and GvHD development. We did not confirm any association of these parameters with GvHD, except for myelodysplastic syndrome diagnosis, where all six patients were found to be negative for GvHD development (Pearson chi-square *p* = 0.025) (Figure 1). Considering the intragroup variability regarding the GvHD+/GvHD− group of patients, several bacterial genera were differentially abundant.

Before HSCT, the gut mucosa layers associated with *Actinomyces* (*p* = 0.024) and *Rumunicoccus torques* (*p* = 0.039) were found to be more abundant in females compared to males, suggesting that females could suffer more from a thinner mucus layer, caused by mucus-degrading *Ruminococcus torques*; in these cases, the development of actinomycosis is usually associated with the break-down of the mucosal barrier. Beneficial, short-chain fatty acid (SCFA)-producing bacterial genera *Dorea* and *Eubacterium hallii* prevailed in the guts of patients with normal BMI (18.5–24.9) compared to low BMI (<18.5) (*p* = 0.012). Regarding the age of patients, the significantly lower relative abundance of *Enterococcus* (*p* = 0.034) and higher relative abundance of *Peptostreptococcaceae* (*p* = 0.025) in the guts of patients older than ten years was determined.

One week after HSCT, *Streptococcus* (*p* = 0.006) abundance was typical for patients with low BMI, while the abundances of *Abiotrophia* (*p* = 0.037) and *Veillonella* (*p* = 0.041) were significant for patients younger than ten years of age.

After one month from HSCT, the *Staphylococcaceae* family prevailed in the gut of patients older than ten years (*p* = 0.033). There were no complex changes or differences between patients based on diagnosis, therapy, BMI, sex, or age. However, several opportune pathogen bacterial genera, like *Enterococcus* (younger than ten years), *Actinomyces* (female), *Streptococcus* (low BMI), or *Staphylococcaceae* (older than ten years), were found to dominate in the gut of patients in certain stages of therapy related to HSCT. 

The analysis of anthropometric parameters and clinical diagnoses of all included patients did not reveal any discernible pattern for the development of GvHD, suggesting that although all patients have undergone a conditioning regimen before HSCT, harsh treatment did not unite the gut microbiome, resulting in a pattern typical for GvHD development. Since the number of patients suffering from the same oncology diagnoses was low, except for ALL, we decided to focus our further analysis exclusively on patients diagnosed with ALL. This decision was guided by the need for a more homogenous patient group to draw meaningful conclusions and to ensure that our study’s outcomes would be as relevant and specific as possible to this patient population.

### 2.2. Multiresistant Bacteria in the Gut of Patients with ALL

The detection of multiresistant bacteria within the gut microbiome has become increasingly critical for our understanding of their potential impact on human health. Multiresistant bacteria are defined as bacteria with two or more resistance genes within their genomes. In this study, resistance genes localized either on chromosomes or on plasmids were included.

Gut resistome analyses uncovered intriguing trends among patients with and without GvHD. Notably, we observed a significant increase in multiresistant bacteria one week after HSCT in patients afflicted by GvHD. At the same time, patients without GvHD predominantly hosted known commensal bacteria with multidrug resistances in the initial phase of the HSCT (before HSCT). Strikingly, one month after HSCT, we observed a rise in resistance genes among patients without GvHD, suggesting a window of opportunity for predicting infection risks. Moreover, our comprehensive analyses of gut microbiota composition unveiled the dominance of multiresistant bacteria, further emphasizing their role as critical actors in gut shaping. The gut of GvHD patients was primarily inhabited by multiresistant bacteria *E. faecium*, i.e., mainly *E. faecium* ME3, *E. faecium* ICU-1-2, *E. faecium* KUHS13, and *E. faecium* VVEswe-R. These microorganisms most often possessed a combination of the *msr(C)*, erm(T), *aac(6′)-li*, *dfrG*, and *ant(6)-la* genes (Table 1). A combination of resistance genes was shown in the second most commonly identified multiresistant bacterium, *Klebsiella pneumoniae*, carrying a combination of the *aph(3″)-Ib*, *sul2*, and *aph(6)-ld* genes (Table 1). 

Our investigation also revealed multiresistant commensals *B. fragilis* (two patients), *Rumincoccus gnavus* (one patient), *Enterocloster clostridioformis* (one patient), or *Turicibacter* (one patient) in the gut of non-GvHD patients before HSCT (Table 1). Remarkably, one of the GvHD patients benefited from multiresistant commensal *bacteria [Ruminococcus] gnavus*, while others showed the presence of opportunistic pathogens. Nevertheless, non-GvHD patients also had multiresistant bacteria such as *E. faecium* (three patients), *K. pneumoniae* (one patient), or *S. aureus* (one patient) before HSCT. Still, their presence was no longer confirmed one week after HSCT (Table 1). On the contrary, the GvHD patients showed the existence of these bacteria mostly one week after HSCT. *S. haemolyticus* strains with resistance genes *erm(C)*, *dfrG*, *mecA*, *tet(K)*, *mef(A)*, *mph(C)*, *msr(A)*, *msr(D)*, *qacA*, *aph(3′)-III*, and *blaZ* was detected in one patient’s gut microbiome. *E. faecium* strains were identified in three other patients, and the other one had *Clostridioides difficile* in his gut microbiome (Table 1).

**Figure 2:**
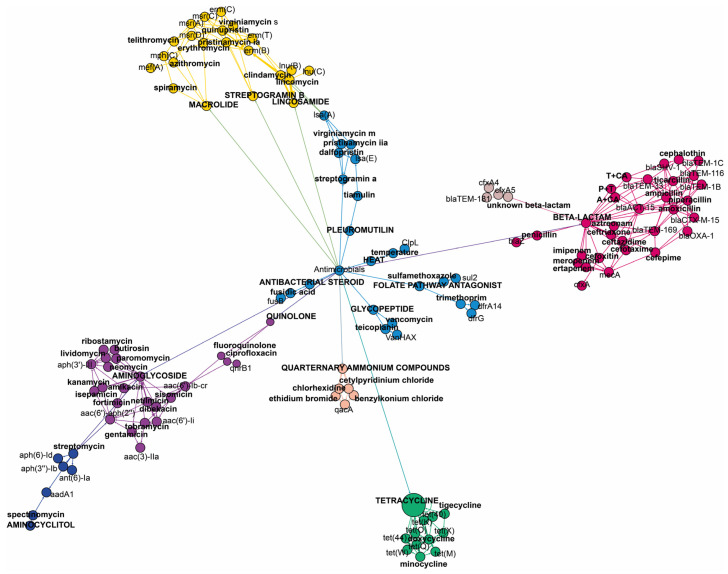
This network shows the relationship between individual resistance genes and antimicrobials. Different colors represent different antimicrobial classes (aminoglycoside—purple; tetracycline—green, macrolide, streptogramin b and, lincosamide—yellow; beta-lactam—pink; quarternary ammonium compounds—beige, unknown beta-lactam—brown), while light blue was used for the class of antimicrobials that were represented with a low number of resistance genes and antimicrobials (glycopeptide, antibacterial steroid, pleuromutilin, folate pathway antagonist, aminocyclitol). P + T—piperacillin + tazobactam. A + CA—ampicillin + clavulanic acid. T + CA—ticarcillin + clavulanic acid.

It is important to note that some of the genes included in this study are multidrug resistance (MDR) genes, i.e., exhibiting resistance to two or more antimicrobials. The *aac(6′)-li* gene is responsible for resistance to dibekacin, gentamicin, netilmicin, or tobramycin from the aminoglycoside family. The *bla-ACT-15* gene confers resistance to amoxicillin, ampicillin, cefotaxime, cefoxitin, and ceftazidime from the beta-lactam family. The *msr(C)* gene provides resistance to erythromycin and telithromycin from macrolides, as well as pristinamycin IA, quinupristin, and virginiamycin S from the streptogramin B class of antimicrobials. Figure 2 shows the specific antimicrobials and antimicrobial classes associated with each identified gene.

### 2.3. Antimicrobials

**Figure 3:**
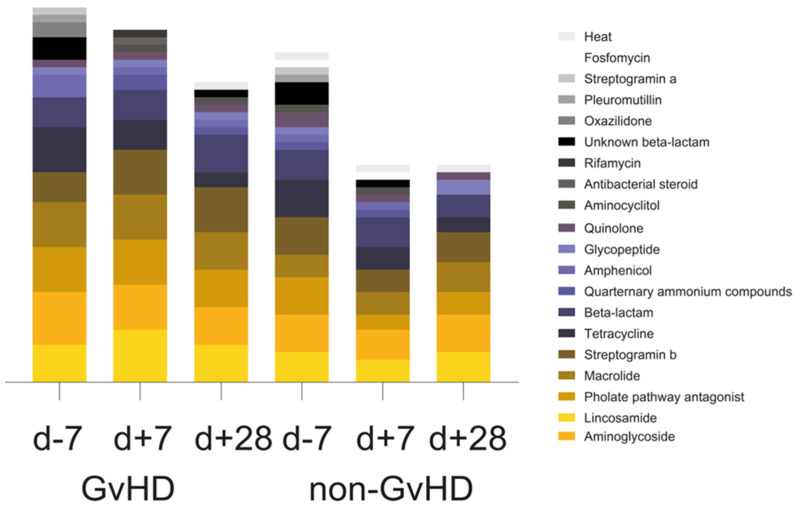
Groups of antimicrobials represented by antimicrobial resistance genes detected in the gut of pediatric oncology patients.

Of the twenty observed groups of antimicrobials, the most prevalent one in the gut microbiome of 67% of GvHD patients before HSCT, 100% of patients one week after HSCT and one month after HSCT, 80% of non-GvHD patients before HSCT, 20% of patients one week after HSCT, and 40% of patients one month after HSCT was aminoglycoside. Furthermore, another three groups of antimicrobials were highly abundant: lincosamides, folate pathway antagonist, macrolides, and beta-lactams. Lincosamide resistance genes were identified in 50% of GvHD patients’ gut microbiomes before HSCT, 50% of patients after one week, and 84% of patients one month after HSCT. The resistance genes from this group were also detected in the gut microbiome of 40% of patients without GvHD before HSCT, 80% of patients after one week, and 60% one month after HSCT. Folate pathway antagonist was detected in 84% of GvHD patients’ gut microbiomes before and one week after HSCT, while it was only detected in 67% of patients one month after HSCT. Its presence was detected in the gut microbiome of 40% of non-GvHD patients before HSCT and three patients one month after HSCT. Macrolide resistance genes were found in the gut microbiome of 34% of GvHD patients and 40% of non-GvHD patients before and 84% of GvHD and 40% of non-GvHD patients one week after HSCT, and finally, in 67% of GvHD patients and 100% of non-GvHD patients one month after HSCT. Tetracyclines were detected in 84% of GvHD patients’ gut microbiomes before HSCT and 60% of non-GVHD patients, while its presence decreased to 34% of GvHD and 40% of non-GvHD patients one week after HSCT and 17% of GvHD patient one month after HSCT. Streptogramin b resistance genes were detected in the gut microbiome of (GvHD/non-GvHD) 34%/40% patients before HSCT, 84%/40% patients one week after HSCT, and 84%/80% one month after HSCT. Before HSCT and one week after HSCT, five patients, 50% of the GvHD, and 40% of the non-GvHD group were potentially resistant to beta-lactams, as were 50% of GvHD patients together with 40% of non-GvHD patients one month after HSCT (Figure 3). The chi-square test showed a significantly positive association between beta-lactam resistance and GvHD outcome (*p* = 0.041). Of the 14 healthy controls, only eight carried resistance genes primarily for tetracycline (*tet(Q)*, *tet(O)*, *tet(W)*), while three controls carried resistance genes for also beta-lactams (*cfxA*). Folate pathway antagonist (*sul2*) was found in one healthy individual (Figure 3).

### 2.4. Resistance and Virulence Genes of the Gut Microbiota of Pediatric Oncology Patients Undergoing HSCT

The ResFinder identified 85 resistance genes across the samples. The resistance gene *aac(6′)-Ii* (aminoglycoside) was the most frequently found gene in the following patient groups: 60% of patients without GvHD and 17% of with GvHD before HSCT, 50% of GvHD and 40% of non-GvHD patients one week after HSCT, and 40% of patients without GvHD and 50% of patients with GvHD one month after HSCT. 

In GvHD patients before HSCT, *sul2* was the most frequently detected resistance gene in the gut microbiome, found in 67% of patients, compared to only 20% of non-GvHD patients. The *tet(W)* gene was also detected in the same percentage of patients before HSCT. *aph(3‴)-lb* was identified in three GvHD and one non-GvHD patient before HSCT, while *blaTEM-1B* was found only in three GvHD patients before HSCT. *dfrg* was identified only in 67% of GvHD patients one week after HSCT in the gut microbiome, while *erm(T)* was detected in only 60% of non-GvHD patients.

We found that antibiotic resistance genes *ant(6)-la* and *msr(C)* were present in the gut microbiome of 50% of patients with GvHD, and *msr(C)* was detected in 40% of non-GvHD patients, while *ant(6)-la* was found in only 20% of non-GvHD patients. After a month of HSCT, a similar number of resistance genes were detected in all patient samples. *msr(C)* and *ant(6)-la* were found in both groups of patients, while *aph(3)-III* with *erm(B)* was detected in 33% of patients with GvHD and 40% of patients without GvHD. The *dfrG* gene was present in 50% of patients with GvHD and 40% without GvHD; it was significantly (*p* = 0.019) more often detected in GvHD patients. The erm(T) gene showed a trend (*p* = 0.074) of being more common in patients with GvHD.

The patients who did not develop GvHD were found to be positive for *Enterococcus faecalis* isolates identified before HSCT. Among these patients, one was positive with two isolates: *E. faecalis* 28157 and WE0851. *E. faecalis* 28157 was found to carry five virulence genes, i.e., endocarditis-specific antigen (*efaA*), sugar-binding transcriptional regulator (*LacI* family, *bopD*), endocarditis and biofilm-associated pilus tip protein (*EbpA*), as well as its major (*ebpC*) and minor (*ebpB*) subunits. On the other hand, the isolate WE0851 carried three identified virulence genes: undecaprenyl diphosphate synthase (*cpsA*), phosphatidate cytidylyltransferase (*cpsB*), and surface protein *Fss1* fibrinogen-binding protein (*fss1*). One GvHD patient carried virulence gene *pcr4*, found in the type III secretion system protein of the *Pseudomonas aeruginosa* strain PA59 one week after HSCT, and in another patient with GvHD, *E. faecium* carried the virulence gene *acm* coding for collagen adhesin precursor. The same gene was identified in 33% of GvHD patients before HSCT and 50% of patients one week after HSCT. Notably, all of these *E. faecium* strains, except for that found in one patient, also harbored the resistance gene *aac(6′)-Ii*, which was found on the chromosome.

### 2.5. Multidrug-Resistant Commensal Bacteria of Gut of Pediatric Patients Suffering from Acute Lymphoblastic Leukemia during HSCT 

In the gut microbiome of GvHD patients, we detected several opportune pathogens and commensals that carry resistance genes in their genomes. However, *Ruminococcus gnavus* NBRC 114413 with resistance genes *catP* and *tet(O)*, *Bacteroides fragilis* DCMOUH0017B with resistance genes *cfxA*, *tet(Q)* and *qnrD1*, and *Parabacteroides distasonis* ATCC 8503 were found in non-GvHD patients. Additionally, *Lachnospiraceae* bacterium strains also carried resistance gene *tet(W)* only in GvHD patients before HSCT. In GvHD patients, we detected only one commensal beneficial bacterium and *Latilactobacillus curvatus* with *ClpL* resistance gene one week after HSCT. 

**Figure 4:**
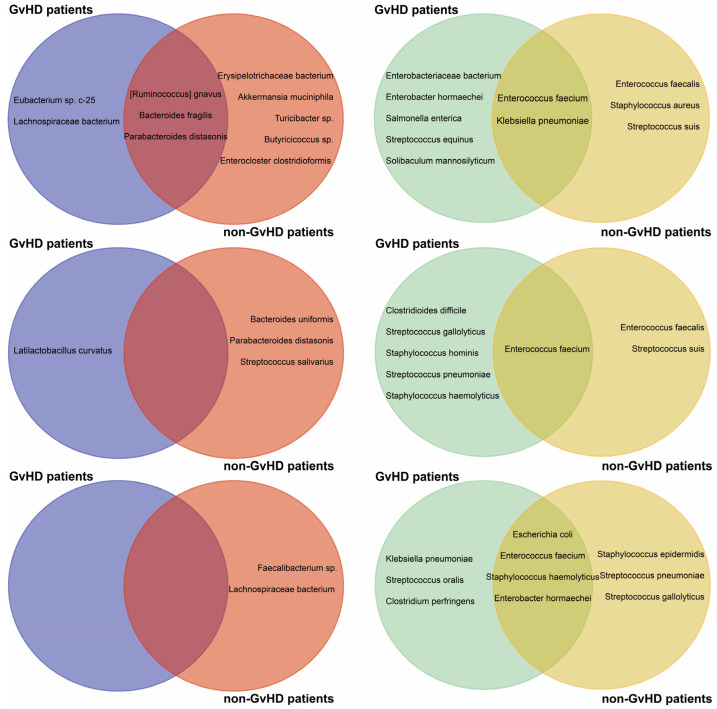
Schematic visualization of multidrug-resistant commensal and opportune pathogen bacteria identified in the gut microbiome of pediatric oncology patients suffering from acute lymphoblastic leukemia developing GvHD. d − 7—before HSCT, d + 7—1 week after HSCT, d + 28—1 month after HSCT.

On the other hand, non-GvHD patients had a higher frequency of detection of resistance genes in their commensal bacteria. *Erysipelotrichaceae* bacterium LH1062 with *tet(44)* gene was detected together with *Akkermansia muciniphila* Akk2670 (*lnu(C)*), the gut barrier protector, before HSCT. Before HSCT, *Turicibacter* sp. H121 (*mef(A)*, *msr(D)*) was also detected. Resistance genes were detected in *Bacteroides uniformis* NBRC 113350 (*tet(Q)*), *B. fragilis* (*cfxA5*), and *Streptococcus salivarius* (*tet(M)*) one week after HSCT. *Faecalibacterium* sp. IP-3-29 (*aph(3′)-III*) and *Lachnospiraceae* bacterium (*tet(W)*) were abundant bacteria carrying resistance genes one month after HSCT. Nevertheless, patients with resistant or multiresistant commensal bacterial strains or isolates often harbored opportune pathogen bacterial strains such as *E. faecium*, *S. haemolyticus*, or *K. pneumoniae*. Remarkably, only one patient who did not develop GvHD did not show resistance carried by non-pathogenic bacteria (Figure 4). 

In the healthy controls, resistance genes were present exclusively in beneficial commensal bacteria, namely, *B. fragilis* with *tet(Q)* and *cfxA* genes, *Phocaeicola dorei* with *tet(Q)* gene, and *P. distanosis* with *tet(Q)* and *cfxA* genes, as well as *Cutibacterium* acnes with *tet(W)* gene. 

### 2.6. Multidrug-Resistant Opportune Pathogens in the Gut of Pediatric Patients Suffering from Acute Lymphoblastic Leukemia during HSCT 

The non-GvHD patients also had detectable *E. faecium* KUHS13 (*dfrG*, *msr(C)*, *ant(6)-la*) and VVEswe-R (*msr(C)*, *aph(3′)-III*, *erm(B)*, *aac(6′)-aph(2″)*) at various timepoints. Additionally, *K. pneumoniae* with resistance genes *aph(3″)-Ib*, *aph(6)-Id*, and *sul2* was detected in these patients prior to the HSCT procedure. Both bacterial species were detected in GvHD and non-GvHD patients before HSCT. MDR *E. faecium* was detected in 60% of non-GvHD but only in 17% of GvHD patients before HSCT and 67% of GvHD patients one week and one month after HSCT. In non-GvHD patients, these species were detected in the gut only one month after HSCT.

Specifically, patients with GvHD were identified with *Enterobacter hormaechei* (*bla*ACT-15 and *qnrB1*), *Enterobacteriaceae* bacterium (*blaTEM-1B* and *dfrA14*), *Salmonella enterica* (*aph(3″)-Ib*, *aph(6)-Id*, *dfrA14*, *sul2*), *Solibaculum mannosilyticum* (*tet(W)*), and *Streptococcus equinus* (*lnu(C)*) prior to HSCT, while non-GvHD patients had *Enterococcus faecalis* (*erm(B)*), *Staphylococcus aureus* (*lnu(B)* and *lsa(E)*), and *Streptococcus suis* (*ant(6)-la*). One week after HSCT, only *E. faecium* with various combinations of *msr(C)*, *aac(6′)-li*, *erm(B)*, *ant(6)-la*, *dfrG*, *VanHAX*, *erm(T)* and *aph(3′)-III* resistance genes were detected in both groups.

**Figure 5:**
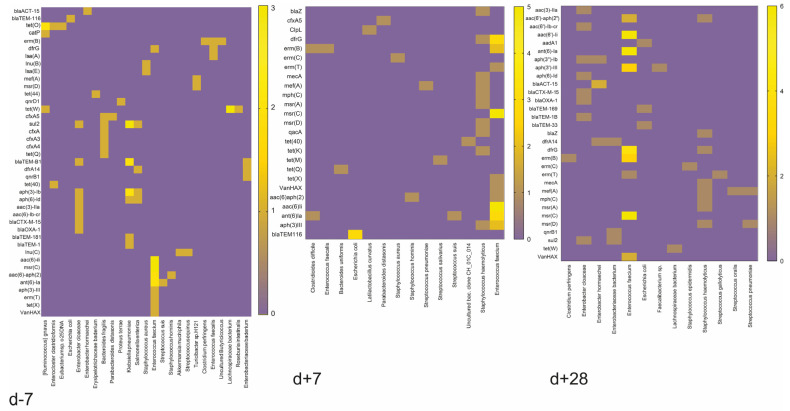
Schematic heatmap visualization of antimicrobial-resistance gene profiles identified in MDR bacteria of all patients. The color represents the number of patients with identified antimicrobial resistance genes carried by specific gut bacteria.

The patients who did not develop GvHD remained resistant even one week after HSCT. However, those who did develop GvHD had an increased relative abundance of opportunistic bacteria such as *S. haemolyticus*, *Clostridioides difficile*, *Streptococcus gallolyticus*, *Staphylococcus hominis*, and *Streptococcus pneumoniae* present in their system. One month after HSCT, both GvHD and non-GvHD patients had *E. faecium*, *S. haemolyticus*, *E. hormaechei*, and *Escherichia coli* present in their system. *K. pneumoniae* and *S. suis* were specific to GvHD patients, while *Staphylococcus epidermidis*, *S. gallolyticus*, and *S. pneumoniae* were specific to non-GvHD patients one month after HSCT. We investigated the antimicrobial-resistance gene profiles of MDR bacteria, concluding that before HSCT most patients carried resistance genes *sul2*, *blaTEM-1B*, *aph(3)-Ib* by *Klebsiella pneumoniae*, *aac(6)-Ii*, *msr(C)*, *aac(6)-aph(2)*, and *ant(6)-Ia* by *Enterococcus faecium*, while one week after HSCT, as well as one month after HSCT, *Enterococcus faecium* carrying *aac(6)-Ii*, *ant(6)-Ia* and *drf(G)* dominated unequivocally (Figure 5).

These results highlight the differences in microbiome composition and antibiotic resistance gene profiles (Figure 5). The unique combination of bacterial strains and resistance genes in each patient may affect their response to antibiotic treatment and the development of GvHD. However, the results showed more variability in gene representation one week after HSCT in GvHD patients. This could be a marker and a timepoint for the detection and determination of the risk associated with GvHD development.

### 2.7. The Gut Bacteriome of Pediatric Oncology Patients Characterized by the 16S rRNA Analysis 

The most abundant bacterial phylum identified by 16S rRNA sequencing was *Bacillota*, with an abundance of 69% in patients with GvHD and 79% in patients without GvHD before HSCT. Patients with GvHD showed a trend of increased relative abundance of *Bacillota* one week (71%) and one month (86%) after HSCT. In contrast, patients without GvHD showed different patterns with decreased relative abundance (72%) one week after HSCT and a significant, faster, and more remarkable increase (99%) one month after HSCT. 

Interestingly, for patients with GvHD, the relative abundance of the second most abundant phylum, *Bacteroidota*, decreased from 19% before HSCT to 3% one week and 0.5% one month after HSCT. However, patients not developing GvHD possessed a relatively stable relative abundance of *Bacteroidota* before HSCT (11%) and one week after HSCT (12%), while a significant decrease (0.4%) could be observed one month after HSCT. 

*Pseudomonadota* was found to be more prominent in patients who developed GvHD (graft-versus-host disease). Prior to HSCT (hematopoietic stem cell transplantation), the abundance of *Pseudomonadota* was 12% in patients with GvHD and 8% in those without GvHD. One week after HSCT, patients with GvHD showed a significant increase in the abundance of *Pseudomonadota*, which reached 23%, whereas patients without GvHD maintained an abundance of 8%. However, one month after HSCT, the abundance of *Pseudomonadota* decreased to 11% in patients with GvHD and 1% in those without GvHD.

Regarding *Verrucomicrobiota*, which had an abundance of less than 1% in all three time points for patients developing GvHD, despite the same abundance before HSCT in the cohort of non-GvHD patients, one week after HSCT, its abundance increased to 8%, suggesting its protective activity of the gut mucus layer. 

**Figure 6:**
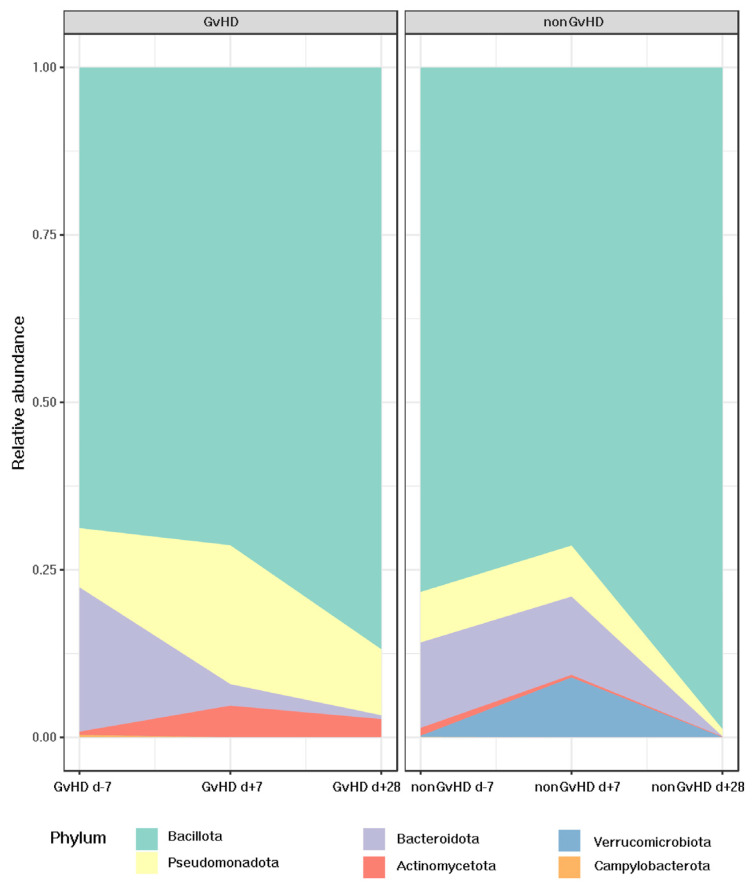
The gut microbiome of patients with acute lymphoblastic leukemia developing GVHD and the relative abundances of bacterial phyla.

*Actinomycetota* was present in all timepoints for both groups, with an abundance of 0.5% before HSCT for patients with GvHD and 1% for patients without GvHD. However, one week after HSCT, there was an increase in the relative abundance of *Actinomycetota* in GvHD patients (4%), while the percentage for patients without GvHD decreased to 0.3%. One month after HSCT, the abundance of *Actinomycetota* in patients without GvHD reached only 0.07%, whereas it was elevated to 2% in the gut of patients with GvHD. *Campylobacterota* was present only before HSCT in both groups, with an abundance of less than 1%. The other bacterial phyla did not show any significant change in relative abundance. (Figure 6).

*Enterococcaceae* was found to be the most abundant family across all timepoints in both groups. In the cohort of patients with GvHD, it had an abundance of 25% in all three timepoints, while in patients without GvHD, it showed an increase in abundance over time, i.e., 33% before HSCT, 52% one week after HSCT, and 79% one month after HSCT. *Bacteroidaceae* showed abundances of 18% in the gut of patients with GvHD and 10% in patients without GvHD before HSCT, with a decrease one week after HSCT (0.4%—GvHD+ patients/0.1% GvHD− patients). The abundances of the two groups differed one month after HSCT, where patients with GvHD had an average of only 0.4%, while patients without GvHD had an abundance of 0.03%. *Lachnospiraceae* was the third most abundant family before HSCT in both groups (13%—GvHD+ patients/10%—GvHD− patients), followed by a decrease to 7% one week after HSCT and <1% one month after HSCT in both groups. *Enterobacteriaceae* showed an increasing trend in patients with GvHD, from 10% before HSCT to 12% one week after HSCT, and a decrease to 8% one month after HSCT. On the other hand, patients without GvHD showed a decrease from 0.4% before and one week after HSCT to 0% one month after HSCT. A decreasing trend was observed in *Erysipelotrichaceae*, *Erysipeloclostridiaceae*, *Ruminococcaceae*, and *Veillonellaceae*.

The most abundant genus was Enterococcus across all timepoints in both groups, with stable abundance in all timepoints in patients with GvHD (25%) and with an increasing trend in patients without GvHD (33%/52%/79%). *Bacteroides* decreased in abundance over time for GvHD patients, with abundances of 18% before HSCT, 0.4% one week after HSCT, and 0.3% one month after HSCT. The gut microbiota of patients without GvHD showed a decrease from 11% before HSCT to 0.1% one week after HSCT and 0.03% one month after HSCT. *Erysipelatoclostridium*, together with *Lachnoclostridium*, *Faecalibacterium*, *Blautia*, and *Roseburia*, gradually decreased in both groups. *Staphylococcus* was higher in the samples one week and one month after HSCT in patients with GvHD, where the abundance increased from 0.3% before HSCT to 20% one week after HSCT and 15% one month after HSCT. The abundances in patients without GvHD were 16% before HSCT, 3% one week after HSCT, and 14% one month after HSCT. Similarly, this trend was observed in *Streptococcus*, increasing in patients with GvHD (0.05%/5%/12%) but decreasing in patients without GvHD (7%/0.3%/3%). *Klebsiella* was another bacterial genus enriched in the gut of patients with GvHD (0%/4%/8%) compared to patients without GvHD, where it was not detected. *Veillonella* was more abundant in the gut of patients with GvHD before HSCT, with an abundance of 3%, while at the other timepoints it was under 1%, similar to what we observed at all timepoints for the patients without GvHD.

**Figure 7:**
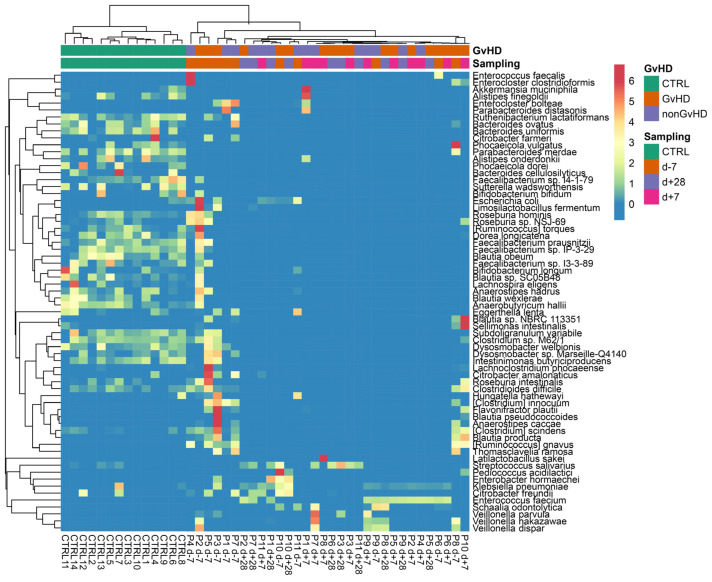
Graphical visualization of the 70 most abundant bacterial species (relative abundance) of the gut microbiome of pediatric oncology patients suffering from acute lymphoblastic leukemia using heatmap.

The most abundant species identified in metagenomic data from both groups was *Enterococcus faecium* at all timepoints. The individual patients showed a wide range of abundances; therefore, a heatmap was used to better visualize the portfolio and the spectrum of individual bacterial species (Figure 7). 

The following dominant pathobionts were detected: *E. faecium*, *E. faecalis*, *E. bolteae*, *S. haemolyticus*, *P. aeruginosa*, and *K. pneumoniae*. In addition, also commensal bacteria were detected in high abundance, including *Akkermansia muciniphila*, *Bacteroides uniformis*, *Parabacteroides distasonis*, *Bacteroides fragilis*, and *Latilactobacillus sakei* (Figure 7).

**Figure 8:**
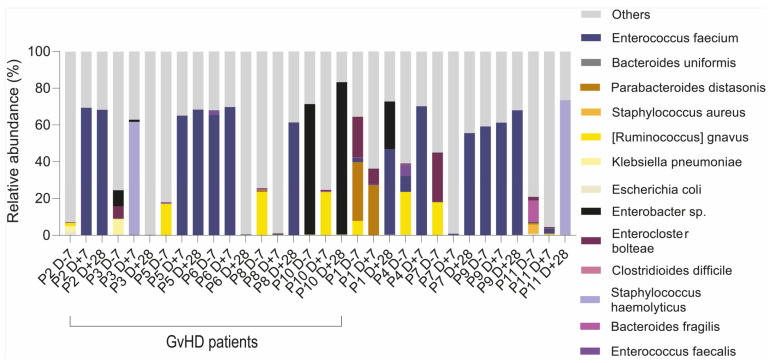
Intestinal dominance of multidrug-resistant bacteria in pediatric patients with acute lymphoblastic leukemia developing GvHD.

We found that multiresistant bacteria were dominant or had a higher abundance within the group of resistant bacterial species (Table 1). For instance, the relative abundance of a multiresistant strain *Ruminococcus gnavus* was 26%, as identified by 16S rRNA analysis and confirmed by whole metagenome sequencing (23%), making it the most abundant bacteria in patients’ gut microbiota. For patients with dominant multiresistant *S. haemolyticus*, these bacteria reached 60% and 73% of total relative abundance, respectively. An analysis of the gut microbiome revealed the presence of multiresistant bacteria that were not identified by cultivation analysis, except for two patients who were found to be positive both in cultivation analysis of sputum or tonsils swabs as well as sequencing analysis (*E. faecium* KUHS13 and ME3 strains) of the gut. Patients with *Enterobacter* sp. multidrug resistance strains showed only a higher abundance of the genus *Enterobacter*, while the species from this genus were not well identified. Our analysis of the metagenomes revealed the consequences of the multidrug-resistant bacteria and their colonization of the disrupted gut microbiota environment (Figure 8).

### 2.8. Development of Gut Mycobiome of Pediatric Patients Suffering from Acute Lymphoblastic Leukemia during HSCT Treatment

The median abundance of *Fungi* found in the samples was 0.03%. The highest abundance was 4.6%, while the lowest was 0.001% (with a standard deviation of 0.73%). The relative abundance was recalculated, with the final abundance of *Fungi* being 100%. 

**Figure 9:**
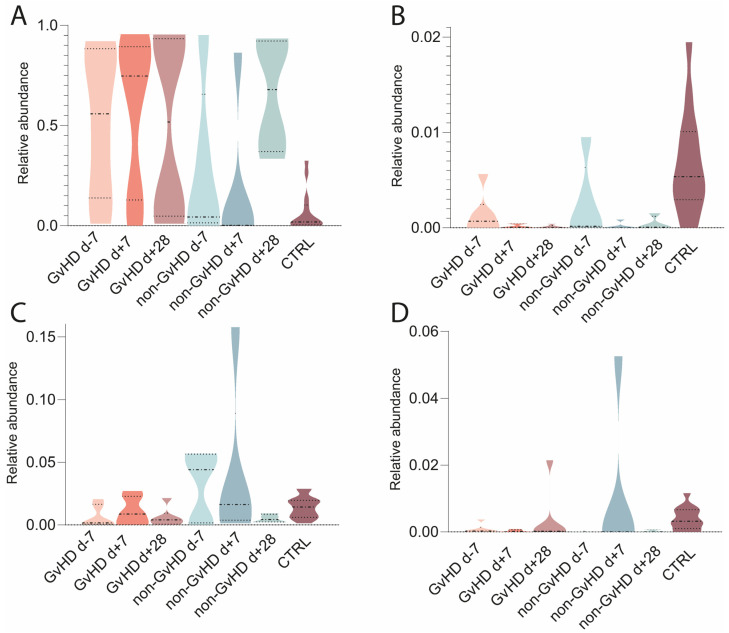
Violin plots showing the relative abundances (%) (recalculated to the Fungi—100%) of selected fungal species identified within the gut of patients developing GvHD. The median with the first and third quartile are marked. (**A**)—*Saccharomyces cerevisiae*; (**B**)—*Malassezia restricta*; (**C**)—*Nakaseomyces glabratus*; (**D**)—*Yarrowia lipolytica*.

The mycobiome was characterized from metagenomic data; the most abundant fungi found were *Saccharomyces cerevisiae,* with average abundances of 44%/50%/50% in GvHD patients and 28%/18%/65% in non-GvHD patients (Figure 9). Healthy controls had an abundance of 7%. The second most abundant fungi in the gut microbiota of patients with GvHD before HSCT was *Yarrowia lipolytica,* with an abundance of 3%, which decreased to less than 1% one week and one month after HSCT. In patients without GvHD, the abundance was less than 1% at all three time points, while healthy controls had an abundance of 1%. *Nakaseomyces glabratus* (*Candida glabrata*) and *Malassezia restricta* (Figure 9) were identified with abundances of 0.1% and 3%, respectively, in GvHD patients. The abundance of *N. glabratus* increased to less than 0.1% one week after HSCT and one month after HSCT. In non-GvHD patients, the abundance of *N. glabratus* increased from less than 1% before HSCT to 2% a week after HSCT and 13% a month after HSCT (Figure 9). The abundance in healthy individuals was less than 1%. *M. restricta* in patients with GvHD increased to 2% one week after HSCT and 5% one month after HSCT. The abundances in patients without GvHD were 3%/8%/1%, while healthy controls had less than 1%. Other abundant species found in patients were *Fusarium keratoplasticum* (0.3%/0.1%/0.01% in GvHD patients and 0.1%/0.1%/0.1% and 1% in healthy controls), *Candida orthopsilosis* (0.1%/0.1%/6% in GvHD patients; 1%/0.1%/0.0003% in non-GvHD patients; 1% in healthy controls), *Candida dubliniensis* (0.1%/0.04%/0.2% in GvHD patients; 2%/2%/0.5% in non-GvHD patients; 1% in healthy controls), and *Neurospora crassa* (0.3%/0.2%/0.6% in GvHD patients; 1%/0.1%/0.6% in non-GvHD patients; 2% in healthy controls). 

Overall, there were no significant differences in the relative abundance of the most abundant fungal species. A statistical analysis allowed us to identify *Marasmius* (*p* = 0.004) as the only differentially abundant fungus that was significantly more abundant in the gut of GvHD patients.

### 2.9. The Alpha Diversity of the Gut Microbiome of Patients with GvHD

**Figure 10:**
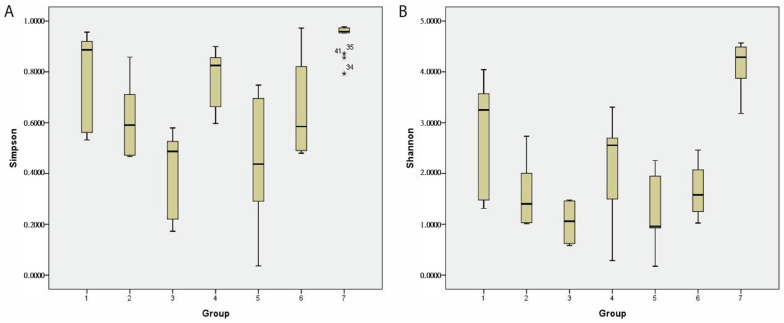
The alpha diversity of the gut microbiome of pediatric patients who have acute lymphoblastic leukemia is represented by Simpson’s (**A**) and Shannon’s (**B**) indices. 1—GvHD patients d − 7; 2—GvHD patients d + 7; 3—GvHD patients d + 28; 4—non-GvHD patients d − 7; 5—non-GvHD patients d + 7; 6—non-GvHD patients d + 28; 7—healthy controls; *—outlier.

The α-diversity was evaluated using the Shannon and Simpson indices. In the cohort of patients developing GvHD, a decreasing trend in diversity richness and evenness was observed. In contrast, patients without GvHD showed a repeated diversity increase one month after HSCT. The Shannon index (average ± stdev) was determined to be 2.7 ± 1/1.6 ± 0.6/1.3 ± 0.8 in GvHD patients and 2.1 ± 0.7/1 ± 0.5/1.7 ± 1 in non-GvHD patients. The Simpson index was determined to be 0.8 ± 0.2/0.6 ± 0.1/0.5 ± 0.2 in GvHD patients and 0.7 ± 0.2/0.4 ± 0.1/0.6 ± 0.3 in non-GvHD patients (Figure 10). The alpha diversity of the gut microbiome of healthy children was significantly (*p* < 0.01) higher (Shannon index 4.1 ± 0.4; Simpson index 0.9 ± 0.1) than that of oncology patients, both either developing or not GvHD. However, no significant changes in diversity between patients developing GvHD and non-GvHD patients were observed, either before or after HSCT treatment. 

Nevertheless, we found that the richness of the gut microbiome in GvHD patients one month after HSCT was significantly lower compared to that before HSCT (*p* = 0.037) and one week after HSCT (*p* = 0.002).

## 3. Discussion

We conducted a metagenomic analysis to investigate the gut resistome of pediatric patients undergoing HSCT and healthy controls and to observe changes in the gut microbiome composition and diversity. As far as we know, the only study that analyzed the gut resistome of GvHD patients was conducted by D’Amico et al. (2019), who found a broad pattern of resistance classes and post-HSCT expansion of the gut resistome in eight pediatric patients [12]. Our study specifically focused on patients with ALL, of whom six had TBI, and five had a chemotherapy-based conditioning regimen. We found that all the factors and treatments that patients routinely undergo can significantly impact microbial composition and diversity. *Klebsiella*, *Enterococcus*, *Staphylococcus*, and *Enterobacter* are part of the human gut microbiota and represent infectious agents. Patients are more susceptible to these bacteria [13]. Several studies have described *Enterococcus* as the main biomarker of GvHD development [8,14,15]. However, we could not confirm this in our study. On the contrary, we found that *Enterococcus* abundance was higher in non-GvHD patients. This could be due to the limited number of patients in our study, whereas other studies had significantly larger cohorts. Our analysis suggests that *Enterococcus* may pose a greater risk to younger children, as its abundance was significantly higher in children under ten years of age. Devaux et al. (2020) described a possible link between GvHD, lactose accumulation in the gut lumen, Reg3 reduction, and lactose auxotroph *E. faecium* proliferation. Due to antimicrobial treatment, depletion in gut microbiota reduced Actinobacteria, which cross-fed the butyrate-producing bacteria from the *Clostridia* class and decreased the metabolism of acetate to butyrate [13]. Butyrate has been described as a protector in GvHD development. All these factors can lead to *Enterococcus* dominance and inflammatory processes, with higher expression of myosin light chain kinase 210 (MLCK210) and subsequent disruption of the intestinal barrier and translocation of bacteria from the gut. Even if it was not found to be multi-drug resistant, *Enterococcus* was found to be the dominant bacteria in most patients.

The gut resistome plays an essential role in medical research, especially for immunocompromised patients [14]. A comparison of the resistance genes present in HSCT patients’ gut microbiome versus healthy controls showed that HSCT patients exhibited a more frequent and broader spectrum of resistances. Moreover, pre-HSCT samples demonstrated a broad spectrum of resistance genes, likely due to exposure to the hospital environment and previous high-dose chemotherapy treatments [15]. Pediatric patients demonstrated an especially pronounced expansion of the gut resistome one week after HSCT in GvHD patients. Since patients undergoing HSCT require immunosuppressive therapy, antimicrobial treatment is often necessary to reduce the risk of infection. However, antibiotics can exert selective pressure, favoring the expansion of antibiotic-resistant strains within the gut microbiota. Despite the use of broad-spectrum antibiotics before HSCT (d − 10), multiresistant bacteria, often with multidrug resistance genes, can survive the combined antimicrobial treatment and lead to therapeutic challenges. Hence, the presence of multidrug resistance genes in the gut microbiota can make common antibiotics less effective.

Our study has highlighted the presence of multiresistant bacteria in the gut microbiota of patients, while the healthy controls showed minimal presence of resistance genes in their gut microbiome. Patients’ gut microbiota also exhibited resistance genes before HSCT due to a strict antibiotic treatment [16]. Resistance genes, which are suggested to be beneficial for our bodies, were detected in commensal bacteria of non-GvHD patients. *Bacteroidales* representatives were most common, but *Lachnospiraceae*, *Clostridiaceae*, *Erysipeltorichales*, and *Akkermansia* were also detected. *Akkermansia* is a bacterial genus often correlated with maintaining the gut barrier and protecting the gut [17]. The gut barrier is especially important in GvHD, where its disruption can lead to the translocation of bacteria into the bloodstream, increasing the risk of infection development. Evidence suggests that conditioning regimen-induced gut barrier damage is the primary driver for GvHD development [18,19,20]. Although commensal bacteria play an essential role in maintaining gut homeostasis and balance, carrying resistance genes could pose a risk for the horizontal transfer of bacterial genes to pathogenic bacteria within the gut or opportunistic pathogens from external sources [21]. Therefore, while the presence of resistance genes in commensal bacteria may not pose an immediate and severe threat, it does highlight the need for careful monitoring and a deeper analysis of the dynamics of the gut microbiome.

MDR bacteria have developed resistance to multiple antibiotics, which makes them more potent in the unfavorable environment created by antimicrobial prophylaxis and conditioning regimens. The colonization of the human body by MDR bacteria could be the most common source of infectious microorganisms; this has been observed to occur more frequently in GvHD patients [22]. Another study found that infection by MDR gram-negative bacteria significantly reduced overall survival one year after HSCT. While MDR bacteria pose a potential danger to patients, deaths associated with MDR bacteria are rare. However, they still contribute to complicated and prolonged hospitalizations. The mechanism by which opportunistic pathogens can affect GvHD development is still unclear. Still, some studies have suggested that these pathogens in the human gut microbiome are prone to acquiring resistance and surviving rigorous antimicrobial therapy. There are supporting hypotheses that may explain how opportunistic pathogens can support GvHD development. One possibility is gut barrier disruption, as some bacterial pathogens can impair gut barrier functions, weaken tight junctions by disrupting zonula occludens, occludin, and claudin, and initiate inflammation [23]. Inflammation is a second possible mechanism, as it can stimulate host cells through microbe-associated molecular patterns, which could enhance inflammation in GvHD [24,25].

Our investigation has revealed important findings that suggest the need for more effective strategies to prevent infection and colonization by multiresistant bacteria. We observed that the combination of broad-spectrum targeted antimicrobial treatment with broad-spectrum antibiotics may not be sufficient to avoid the presence of multiresistant bacteria, which were dominant or significantly more abundant in individual patients. Furthermore, this combination of antibiotics was associated with a loss in diversity and an enrichment in the presence of gut bacteria with resistance genes. These findings highlight the need for more effective and targeted approaches to managing infections in immunocompromised patients, as well as the importance of monitoring the dynamics of the gut microbiome. We have identified shifts in the gut microbiota composition between GvHD and non-GvHD patients, consistent with previous studies. We identified elevated levels of facultative anaerobes from the *Bacillota* phylum, such as *Enterococcus* and *Staphylococcus*, along with a decrease in *Bacteroidota* [26]. However, the time frame for these changes differed between the two groups. GvHD patients experienced a rapid reduction in *Bacteroidota* abundance just one week after HSCT, while non-GvHD patients experienced this shift one month after transplantation. Additionally, the *Pseudomonadota* phylum showed varying abundance between the two groups, with GvHD patients displaying higher levels. The representants of this phylum were detected in higher abundance in the HSCT patients [27]. Despite these differences, we also noticed a commonality in the loss of SCFA-producing bacteria across all patient groups compared to healthy controls, predominantly from the *Clostridia* class. These findings align with previous research, indicating the consistency of these alterations in the context of HSCT [28,29,30]. These observations have important implications for future research and clinical practice, as they suggest that restoring SCFA-producing bacteria in the gut may be a key strategy in promoting gut health and reducing the risk of complications after HSCT.

Furthermore, our study has identified a substantial decrease in alpha diversity among transplant recipients compared to healthy controls, reflecting the reduced overall diversity in the gut microbiota of these patients. We observed that GvHD patients experienced a more pronounced loss of diversity over time, which may contribute to the pathogenesis of GvHD and other health issues. These findings highlight the need to develop interventions that promote diversity and improve outcomes in transplant recipients. Further research is necessary to identify the factors contributing to decreased diversity in the gut microbiota of these patients and to develop targeted interventions based on these factors. 

The role of the gut mycobiome in acute GvHD has been described as showing involvement of *Candida* spp., primarily represented by *Candida albicans*, in the development of aGvHD with higher severity and more often developed gastrointestinal aGvHD [31]. Not the diversity of gut mycobiome, but *Candida* spp. abundance at day 0 of HSCT has been shown to play a crucial role in the overall survival of patients, as well as GvHD development after alloHSCT, probably triggered by β-d-glucan and mannans, leading to the induction of Th 17 and IL-23 response in the mucus layer [32]. Currently, however, only studies focused on adult oncology patients have been published; notably, most of the patients in these studies did not receive antifungal therapy [31,32]. Our results, therefore, provide unique information on the gut mycobiome of pediatric oncology patients suffering from acute lymphoblastic leukemia undergoing HSCT with a focus on GvHD development. Although there was a limited number of patients in our study, *Candida* spp. was represented exclusively by *Nakaseomyces glabratus* (*Candida glabrata*) and *Candida dubliniensis*, but not *Candida albicans*. Both fungal species were more abundant in the gut of patients not developing GvHD either before HSCT or one week after HSCT, suggesting that this represents a biomarker of better HSCT outcome and higher microbiome diversity.

The main limitation of our study was the low number of patients. However, we included all pediatric oncology patients admitted to the hospital during three consequent years, provided they were able to provide a biological specimen for gut microbiome analysis at all three established timepoints. Additionally, so far, there is only one scientific paper focused on resistome profiling of patients with GvHD, so our findings represent unique results that cannot be compared to previously published articles. 

We are aware of the heterogeneity of patients, and that is why we performed analyses on confounding factors that could possibly contribute to patient gut microbiome variability. Even though the subgroups were smaller, we aimed to clearly identify the potential effect of confounding factors and pinpoint their potential role in GvHD outcome. If no significant differences in gut microbiome composition or diversity were observed, we estimated the variability of sample groups without any significance to the measured parameter. 

Regarding the limitations of the bioinformatics tools and databases, a plethora of different approaches are used for microbiome analysis; therefore, we used the most relevant bioinformatic tools and approaches (to the best of our knowledge) for microbiome 16S rRNA and metagenomic analysis, including the Silva database used with QIIME2 and DADA2, as well as Kraken2, with the standard database, including relevant and reliable representative genomes of microorganisms for taxonomical classification. However, we admit that analyses of the other variable regions of 16S rRNA may lead to similar but alternative results. Another possible limitation involved the ResFinder database, which does not cover the resistance mechanisms of chromosomal mutations and only consists of known and described resistance gene sequences. On the other hand, the vfdb database for virulence genes is limited to only 32 bacterial genera classified as pathogens.

We nonetheless believe that our study provides valuable insights into the dynamics of the gut microbiota in transplant recipients and can guide future research and clinical practice in this area.

## 4. Materials and Methods

### 4.1. Subjects

In total, 32 patients were involved in the present study for gut microbiome analysis. Each patient provided one to three biological specimen samples. For paired analyses, 61 fecal samples were obtained from 21 randomly assigned pediatric oncology patients at the Department of Paediatric Haematology and Oncology-Bone Marrow Transplant Unit (Faculty of Medicine at Comenius University and National Institute of Child Diseases) from 2019 to 2021. The samples were collected seven days before HSCT (d − 7), one week (d + 7), and one month (d + 28) after HSCT. 

The cohort of patients included 11 males and seven females from three to 18 years of age (11.8 ± 5.2 years). Eleven patients were diagnosed with acute lymphoblastic leukemia, three with acute myeloid leukemia, two with myelodysplastic syndrome, one with anaplastic large cell lymphoma, and one with X-linked lymphoproliferative disease. Twelve patients developed febrile neutropenia (FN), and nine patients were diagnosed with GvHD; six had been diagnosed with both GvHD and FN. Of the eleven patients (five females and six males) with acute lymphoblastic leukemia, six were treated in a conditioning regimen with total body irradiation (TBI) and five with chemotherapy; five patients developed GvHD. Of the five patients undergoing chemotherapy, three developed GvHD, while three of the six patients with TBI developed GvHD. All patients developed GvHD from day 16 after HSCT to day 35. The legal representatives of all participants signed informed consent. Samples from fourteen healthy children (six males, eight females) aged 4 to 18 (8.5 ± 4.4 years) were collected in 2021. Randomly assigned healthy individuals had not been diagnosed with any acute or chronic disease and had not received any antimicrobial treatment for one month before sampling. The legal representatives of all participants signed informed consent forms. For the purposes of the present study, patients, that provided at least two biological specimens, obtained within the period of HSCT therapy, were included.

### 4.2. V1-V3 16S rRNA Gene Amplicon Library Preparation and Bioinformatic Analysis

DNA was isolated using a Zymobiomics DNA/RNA isolation kit. The nucleic acids were isolated according to the manufacturer’s protocol. DNA was used for V1-V3 16S rRNA amplicon generation and whole-genome shotgun sequencing. The amplicon PCR was prepared using 5× HotFirePOL Mastermix (Solis BioDyne, Tartu, Estonia) with a primer pair from the Human Microbiome Project (https://www.hmpdacc.org/, accessed on 1 November 2023) and a 30-cycle PCR program. The PCR products were then indexed with the low-cycle protocol using the Nextera XT Index kit (Illumina, San Diego, CA, USA). 

The concentration of the final libraries was measured by Qubit 4.0 Fluorometer (Thermo Fisher Scientific, Waltham, MA, USA), and the DNA profile of the obtained sequences was determined with an Agilent Bioanalyzer using DNA High Sensitivity Kit (Agilent Technologies, Santa Clara, CA, USA). The final libraries were sequenced using the Illumina MiSeq platform via300 bp paired-end reads (Illumina, San Diego, CA, USA).

Samples were analyzed using the Qiime2 2022.11 pipeline [33]. Imported paired-end sequences with quality data were corrected and filtered using the DADA2 QIIME2 plugin with the following parameters: --p-trunc-len-f 285, --p-trunc-len-r 285, --p-trunc-q 2, and --p-max-ee-r 3 [34]. Output from DADA2 was used for further taxonomic classification. For the taxonomic classification of the V1-V3 region of 16S rRNA, we used the pre-trained Silva 138 classifier in the q2-feature-classifier function [35,36]. OTUs classified with the Qiime2 2022.11 pipeline were used to create a sample-specific bacterial profile.

### 4.3. Whole-Genome Shotgun Sequencing and Bioinformatic Analysis

DNA libraries were prepared using a Nextera kit based on transposon tagmentation and PCR. For metagenomic analysis, total DNA was fragmented using transposon-based chemistry (Illumina, San Diego, CA, USA); the obtained fragments were further indexed using low cycle PCR with a Nextera XT Index kit according to manufacturer’s instructions (Illumina, San Diego, CA, USA). Final DNA libraries were checked with a Qubit 4.0 Fluorometer (Thermo Fisher Scientific, Waltham, MA, USA) for quantity and chip electrophoresis using an Agilent Bioanalyzer 2100 system (Agilent Technologies, Santa Clara, CA, USA) with a High Sensitivity DNA Kit to assess quality. Samples were sequenced on the Illumina NextSeq (Illumina, San Diego, CA, USA) platform (2 × 150 bp). All steps in the methodology were performed according to the manufacturer’s protocol.

The reads underwent pre-processing, involving quality control and trimming, through an in-house Galaxy web instance [37]. Subsequently, quality reports for the reads were generated using the FastQC tool [38]. Trimming was executed using Trimmomatic—Galaxy Version 0.38.1 with the following parameters: CROP:296, HEADCROP:17, MINLEN:50 [39].

For metagenomic assembly, the metaSPAdes v3.15.4 software was employed, utilizing the default settings [40]. To classify the taxonomical information of the trimmed reads and metaSPAdes scaffolds, Kraken 2 was used in conjunction with a custom-built database. This database incorporated information on archaea, bacteria, plasmids, viruses, humans, fungi, protozoa, and UniVec_core Kraken 2 sequence libraries, which were retrieved on 8 March 2023. The creation of this database followed the Kraken 2 protocol [41].

### 4.4. Identification of the Drug Resistance and Virulence Genes

Sequences assembled to contigs were used as the input for the ABRIcate tool Galaxy version 1.0.1 (https://github.com/tseemann/abricate) with the default settings. The ResFinder database was used for the detection of resistance genes, and the VFDB database was used for virulence gene identification [42]. The minimum DNA identity was 97%, and the minimum DNA coverage was 100%. The presence/absence of a gene expressed as 1/0 was used for further statistical analysis. The individual contigs with detected resistance genes were then analyzed through BLAST (megablast), which allowed the identification of bacterial strains carrying the antimicrobial-resistance genes [43]. The bacterial strains with the highest identity and coverage score were considered. 

### 4.5. Statistical Analysis

Data normality was determined by SPSS (IBM Corp. Released 2012. IBM SPSS Statistics for Windows, Version 21.0. Armonk, NY, USA: IBM Corp.) using the *p*-value of the Shapiro-Wilk test. For further statistical analysis of the differential abundance of bacterial and fungal taxa, the LefSe tool based on the Kruskall-Wallis test was applied [44]. ClustVis tool BETA version (https://biit.cs.ut.ee/clustvis/) was used for heatmap visualization, and Gephi version 0.9.7 (https://gephi.org/) was used for the network visualization of the individual resistance genes for the specific antimicrobials. GraphPad (GraphPad Prism version 8.0.0 for Windows, GraphPad Software, San Diego, CA, USA, www.graphpad.com) was used for other graphical visualizations (bar charts, heatmaps), and the stack-area plot was made using the Microbiome analyst online tool [45].

## Figures and Tables

**Table 1:** List of multidrug-resistant bacteria in the gut of patients undergoing HSCT.

Patient	d − 7		d + 7		d + 28	
GvHD PATIENTS						
P2	*Klebsiella pneumoniae* strain 314013 plasmid p314013-KPC	*blaTEM-181*, *blaTEM-1B*, *blaTEM-1C*	*Enterococcus faecium* KUHS13 DNA	*dfrG*, *msr(C)*, *ant(6)-Ia*	*Enterococcus faecium* KUHS13 DNA	*dfrG*, *msr(C)*, *ant(6)-Ia*
	*Salmonella enterica* subsp. enterica serovar Manhattan strain SA20084699 plasmid unnamed2	*aph(3″)-Ib*, *aph(6)-Id*, *dfrA14*, *sul2*	*Enterococcus faecium* strain ME3	*aac(6′)-Ii*, *erm(T)*	*Enterococcus faecium* strain ME3	*aac(6′)-Ii*, *erm(T)*
P3	*Klebsiella pneumoniae* subsp. pneumoniae strain 21 plasmid pKpQIL_pKpN	*blaTEM-1B*, *aph(3″)-Ib*, *sul2*	*Staphylococcus haemolyticus* strain CGMH-SH51	*dfrG*, *mecA*, *tet(K)*	*Staphylococcus haemolyticus* strain Sh29/312/L2	*mef(A)*, *msr(D)*
			*Staphylococcus haemolyticus* strain Sh29/312/L2	*mef(A)*, *mph(C)*, *msr(A)*, *msr(D)*, *qacA*, *aph(3′)-III*, *blaZ*		
P5			*Enterococcus faecium* strain ME3	*aac(6′)-Ii*, *dfrG*, *msr(C)*, *aac(6′)-aph(2″)*	*Enterococcus faecium* strain ME3	*aac(6′)-Ii*, *dfrG*, *msr(C)*
			*Enterococcus faecium* strain VVEswe-R plasmid pVVEswe-R4	*ant(6)-Ia*, *aph(3′)-III*, *erm(B)*	*Enterococcus faecium* strain VVEswe-R plasmid pVVEswe-R4	*ant(6)-Ia*, *aph(3′)-III*, *erm(B)*
P6	*Enterococcus faecium* KUHS13 DNA	*msr(C)*, *ant(6)-Ia*, *aac(6′)-Ii*, *dfrG*	*Enterococcus faecium* KUHS13 DNA	*msr(C)*, *ant(6)-Ia*, *dfrG*, *aac(6′)-Ii*		
P8	*[Ruminococcus] gnavus* strain NBRC 114413	*catP*, *tet(O)*			*Enterococcus faecium* strain ICU-2-1	*aac(6′)-Ii*, *VanHAX*, *dfrG*
P10	*Enterobacter cloaceae* strain EclC2185 plasmid pEclC2185CTXM15	*aph(3″)-Ib*, *aph(6)-Id*, *blaCTX-M-15*, *blaTEM-1B*, *sul2*, *aac(3)-IIa*, *aac(6′)-Ib-cr*, *blaOXA-1*	*Clostridioides difficile* strain DSM 104450	*ant(6)-la*, *erm(B)*	*Enterococcus faecium* strain VVEswe-R	*msr(C)*, *ant(6)-Ia*, *aph(3′)-III*
					*Enterobacter cloaceae* strain EclC2185 plasmid pEclC2185CTXM15	*aph(3″)-Ib*, *aph(6)-Id*, *blaCTX-M-15*, *blaTEM-1B*, *sul2*, *aac(3)-IIa*, *aac(6′)-Ib-cr*, *blaOXA-1*
non-GvHD PATIENTS						
P1	*Enterococcus faecium* strain HY07	*acc(6′)-Ii*, *tet(X)*			*Enterobacter hormaechei* subsp. steigerwaltii strain 34998 plasmid p34998-210.894kb	*aph(3″)-Ib*, *aph(6)-Id*, *dfrA14*, *sul2*, *blaACT-15*
	*Bacteroides fragilis* strain FDAARGOS	*tet(X)*, *cfxA5*			*Enterococcus faecium* strain ICU-1-2	*ant(6)-Ia*, *aph(3′)-III*
					*Enterococcus faecium* strain VB12993 plasmid pVB12993_p3	*aph(3′)-III*, *acc(6′)-aph(2″)*, *msr(C)*
P4	*Enterococcus faecalis* isolate WE0851	*erm(B)*, *dfrG*, *lsa(A)*				
	*Bacteroides fragilis* strain BFG-569	*cfxA*, *cfxA3*, *cfxA4*, *cfxA5*				
	*Enterococcus faecium* isolate E8440 plasmid: 4	*aac(6′)-Ii*, *ant(6)-Ia*, *aph(3′)-III*, *msr(C)*				
	*[Ruminococcus] gnavus* strain NBRC 114413	*tet(O)*, *tet(W)*				
	*Staphylococcus aureus* strain NX-T55	*lnu(B)*, *lsa(E)*				
P7	*Klebsiella pneumoniae* strain QD23	*aph(3″)-Ib*, *aph(6)-Id*, *sul2*			Enterococcus faecium KUHS13 DNA	*dfrG*, *msr(C)*, *ant(6)-Ia*
	*Enterocloster clostridioformis* FDAARGOS_739	*tet(40)*, *tet(O)*				
	*Turicibacter* sp. H121	*mef(A)*, *msr(D)*				
P9	*Enterococcus faecium* strain VVEswe-R	*msr(C)*, *aph(3′)-III*, *aac(6′)-aph(2″)*, *erm(B)*			*Enterococcus faecium* strain VVEswe-R	*msr(C)*, *aac(6′)-aph(2″)*, *erm(B)*
	*Enterococcus faecium* strain ICU-1-2	*aac(6′)-Ii*, *erm(T)*				
P11					*Staphylococcus haemolyticus* strain CGMH-SH51	*aac(6′)-aph(2″)*, *dfrG*, *mecA*, *mph(C)*, *msr(A)*

## Data Availability

The data represented in this study are openly available in the SRA database under BioProject ID PRJNA1009258.
